# Modeling Pinot Noir Aroma Profiles Based on Weather and Water Management Information Using Machine Learning Algorithms: A Vertical Vintage Analysis Using Artificial Intelligence

**DOI:** 10.3390/foods9010033

**Published:** 2019-12-30

**Authors:** Sigfredo Fuentes, Eden Tongson, Damir D. Torrico, Claudia Gonzalez Viejo

**Affiliations:** 1 School of Agriculture and Food, Faculty of Veterinary and Agricultural Sciences, University of Melbourne, Melbourne VIC 3010, Australia; eden.tongson@unimelb.edu.au (E.T.); damir.torrico@lincoln.ac.nz (D.D.T.); cgonzalez2@unimelb.edu.au (C.G.V.); 2 Department of Wine, Food and Molecular Biosciences, Faculty of Agriculture and Life Sciences, Lincoln University, Lincoln 7647, New Zealand

**Keywords:** wine quality, machine learning modeling, weather

## Abstract

Wine aroma profiles are determinant for the specific style and quality characteristics of final wines. These are dependent on the seasonality, mainly weather conditions, such as solar exposure and temperatures and water management strategies from veraison to harvest. This paper presents machine learning modeling strategies using weather and water management information from a Pinot noir vineyard from 2008 to 2016 vintages as inputs and aroma profiles from wines from the same vintages assessed using gas chromatography and chemometric analyses of wines as targets. The results showed that artificial neural network (ANN) models rendered the high accuracy in the prediction of aroma profiles (Model 1; *R* = 0.99) and chemometric wine parameters (Model 2; *R* = 0.94) with no indication of overfitting. These models could offer powerful tools to winemakers to assess the aroma profiles of wines before winemaking, which could help adjust some techniques to maintain/increase the quality of wines or wine styles that are characteristic of specific vineyards or regions. These models can be modified for different cultivars and regions by including more data from vertical vintages to implement artificial intelligence in winemaking.

## 1. Introduction

Wine quality traits are difficult to assess in a rapid and objective way in vineyards, especially before winemaking. Usually, quality assessments that are performed in the wine industry are related to the acidity and sugar content in berries (Brix or Baume) to assess maturity [1,2]. However, this assessment only gives information about the amount of alcohol and acidity in the final wine through fermentation. Hence, berry sugars/acidity do not provide useful information on any other important quality trait, such as the potential aroma profiles that could be obtained in the final wine.

Alcohol present in beverages has been found to have an effect on the perception of flavor and aromas, as it aids in the release of volatile aromatic compounds [3]. Furthermore, higher alcohol wines have been sometimes regarded as beneficial for the physicochemical expression of color and other quality traits that impact their sensory evaluation [4]. However, increasing the alcohol content in wines is a problem nowadays due to climate change, specifically global warming. Specifically, higher temperatures are compressing phenological stages, resulting in earlier harvest during hotter months around the globe [5,6,7,8]. This phenomenon produces a double global warming effect in grapevines, which can result in berry shrivel with the associated concentration of sugar in berries, and the degradation of color and aroma compounds, which impact the sensory aroma and flavor profile of final wines [7,9]. Recently, the assessment of mesocarp living tissue has been associated with quality traits for different grapevine cultivars for winemaking [10]. Berry cell death starts around 90 days after full bloom; it is a programmed cell death, which can be uncoupled from sugar accumulation and berry shrivel (both exacerbated by higher temperatures) and can determine the final quality of wines, aroma profile, and sensory appreciation [11,12]. Hence, there is a direct link between the seasonal weather characteristics, which are mainly temperature expressed in thermal time (degree days) accumulated over 10 °C and phenological stages occurrence and duration [13], berry cell death, wine quality, and aroma profiles [11,12]. Furthermore, these berry quality traits can be manipulated using different irrigation techniques, such as regulated deficit irrigation (RDI) [14,15,16,17,18,19,20] and partial rootzone drying (PRD) [21,22,23,24,25].

Some methods using proximal remote sensing within the near-infrared (NIR) light spectrum reflectivity have been developed to assess quality traits from berries in a non-destructive way. Some applications have been implemented to assess the sugar content in berries [26,27], berry pigments [28,29], phenolic compounds [30,31], and grape maturity in general [28,32,33,34]. However, since these techniques are still manual, they cannot account for the natural intra-bunch and vineyard spatial variability, requiring a huge number of measurements and modeling strategies to obtain meaningful results.

Other techniques have been developed thanks to recent advances in unmanned aerial vehicles and remote sensing techniques to assess grape maturity, which can take into account within-vineyard variability using high-resolution multispectral imagery analysis [35,36,37]. However, studies have been limited to a few flights per season, and the indirect assessment of berry quality and maturity may hamper results. Furthermore, associated costs for data acquisition, post-processing to obtain orthomosaics, data analysis for classification, and thematic map production are still costly, requiring in many countries licensed pilots and high data analysis power to obtain meaningful models.

This paper presents machine learning modeling strategies applying integrated vineyard weather and irrigation management parameters as inputs and the aroma profiles as targets obtained from a vertical wine library from a boutique vineyard. The results from this modeling strategy could offer an important tool to winemakers to assess the aroma profiles for future vintages before winemaking. The knowledge of potential aroma profiles of the final wine may allow making adjustments within the winemaking to maintain or increase quality traits in the final wine to maintain a specific wine style that is characteristic of the wine region or particular vineyard.

## 2. Materials and Methods 

### 2.1. Study Area and Weather/Irrigation Management Data Acquisition

**Figure 1:**
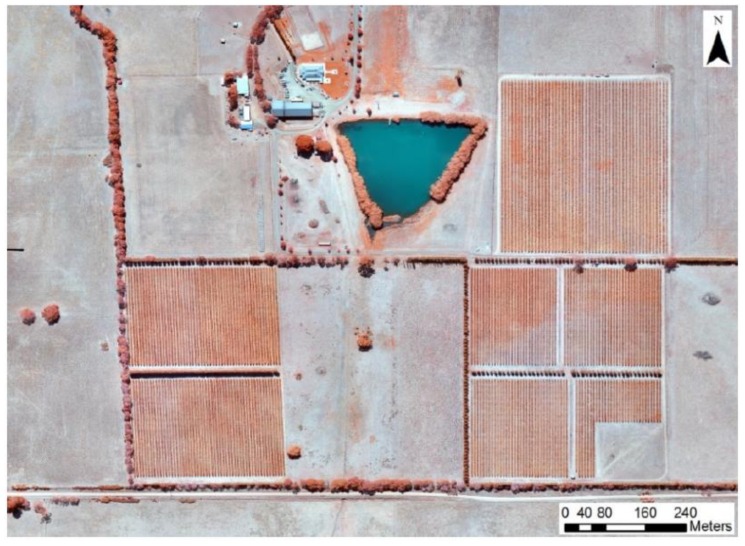
Aerial image of the study area obtained using an unmanned aerial vehicle (UAV) in the 2015–2016 growing season from a total area planted of 42 hectares.

The study was conducted using weather and management data and wine samples from a vertical wine library belonging to a commercial vineyard located at an elevation of 540 m.a.s.l in the South of the Great Dividing Range of the Macedon Ranges in the sub-region of Romsey/Lancefield, Victoria in Australia. The vineyard is situated at a distance from the mitigating influence of the ocean (Figure 1), and the cultivars planted consist of 69% Pinot noir, 26% Chardonnay, and 5% Pinot gris, and use mostly the lyre training system. The study was conducted for vertical vintages from 2008 to 2016 of Pinot noir cultivars, and weather/irrigation management data were obtained from the same site for each season. Information such as (i) solar exposure from veraison to harvest (V-H), (ii) solar exposure from September to harvest (S-H), (iii) maximum January solar exposure (MJSE), (iv) degree days from S-H (DD-S-H), (v) maximum January temperature (MJT), (vi) mean maximum temperature from V-H (MeanMaxTV-H), and (vii) mean minimum temperature from V-H (MeanMinTV-H) was extracted from the Bureau of Meteorology (BoM). Furthermore, the water balance (WB) was calculated using the irrigation (I), rainfall (RF), and evapotranspiration (ET_c_) data using the following Equation (1):

(1) $$WB=I+RF\left(0.85\right)-ET_{c}$$

where WB = water balance; I = irrigation applied in megaliter (ML); RF = effective rainfall, considering 85% of the water is available to the plant, and ETc = crop evapotranspiration calculated using the corresponding crop coefficient (Kc) for different phenological stages [14].

### 2.2. Physicochemical Analysis

Wines from each vintage were analyzed in triplicates for the different physicochemical data measured in this study. A volume of 20 mL of each wine sample was poured in a 60 × 15 mm Greiner Bio-One Polystyrene Petri dish (item number 628102; Greiner Bio-One, Kremsmünster, Austria) and placed on a white uniform surface. Color in CIELab and RGB scales was measured using a NIX Pro color sensor (NIX Sensor Ltd. Hamilton, Ontario, Canada). The UV-Vis spectra from 380 to 780 nm were acquired with a Lighting Passport Pro portable spectrometer (Asensetek Incorporation, New Taipei City, Taiwan). To calculate color intensity, the absorbance of 420, 520, and 620 nm were summed, while for color hue, the absorbance from 420 nm was divided by the value from 520 nm. Fifty mL of each wine sample were used to determine liquid density (weight divided by volume), pH was determined using a pH-meter (QM-1670, DigiTech, Sandy, UT, USA), total dissolved solids (TDS) and electric conductivity (EC) were measured with a Yuelong YL-TDS2-A digital water quality tester (Zhengzhou Yuelong Electronic Technology Co., Ltd, Zhengzhou City, Henan Province, China), salt concentration was measured using a digital salt-meter (PAL-SALT Mohr, Atago Co., Ltd. Saitama, Japan), and alcohol content using an AlcolyzerWine M alcohol meter (Anton Paar GmbH, Graz, Austria).

### 2.3. Gas Chromatography–Mass Spectroscopy

A 5 mL sample of each wine replicate was poured into a 20 mL screw cap vial and sealed with an 18 mm magnetic screwcap with a polytetrafluoroethylene and silicone liner. These samples were analyzed with the method proposed by Gonzalez Viejo et al. [38] using a high-efficiency gas chromatograph with a mass selective detector 5977B (GC-MSD; Agilent Technologies, Inc., Santa Clara, CA, USA), coupled with a PAL3 autosampler system (CTC Analytics AG, Zwingen, Switzerland). The GC-MSD has a detection limit of 1.5 fg, and an HP-5MS column was attached (length: 30 m, inner diameter: 0.25 mm, film: 0.25 μ; Agilent Technologies, Inc., Santa Clara, CA, USA), while the flow rate was set to 1 mL min^−1^ of the carrier gas (Helium). Headspace with solid-phase microextraction (SPME) and a divinylbenzene–carboxen–polydimethylsiloxane grey fiber (1.1 mm; Agilent Technologies, Inc., Santa Clara, CA, USA) was used. Incubation time was set to 20 at 45 °C with a 5 min cycle and 1 min for fiber conditioning (170 °C). Furthermore, the extraction time was set to 40 min with agitation. Two blank samples were used, one at the start and one at the end to avoid any carryover effect. To identify the volatile compounds, the National Institute of Standards and Technology library (NIST; National Institute of Standards and Technology, Gaithersburg, MD, United States) was used. Only the compounds with ≥ 80% certainty were reported.

### 2.4. Statistical Analysis and Machine Learning Modeling

**Figure 2:**
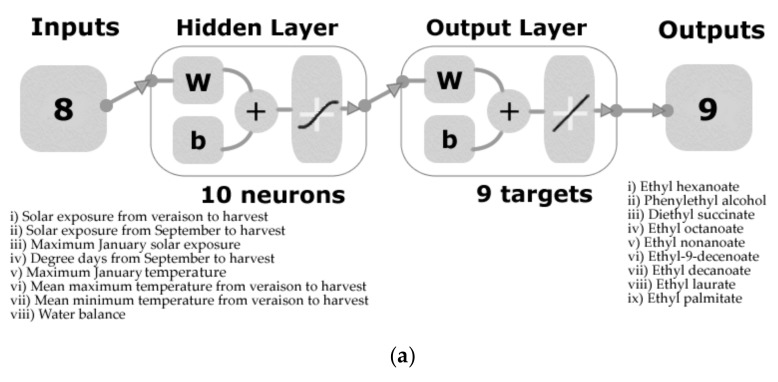

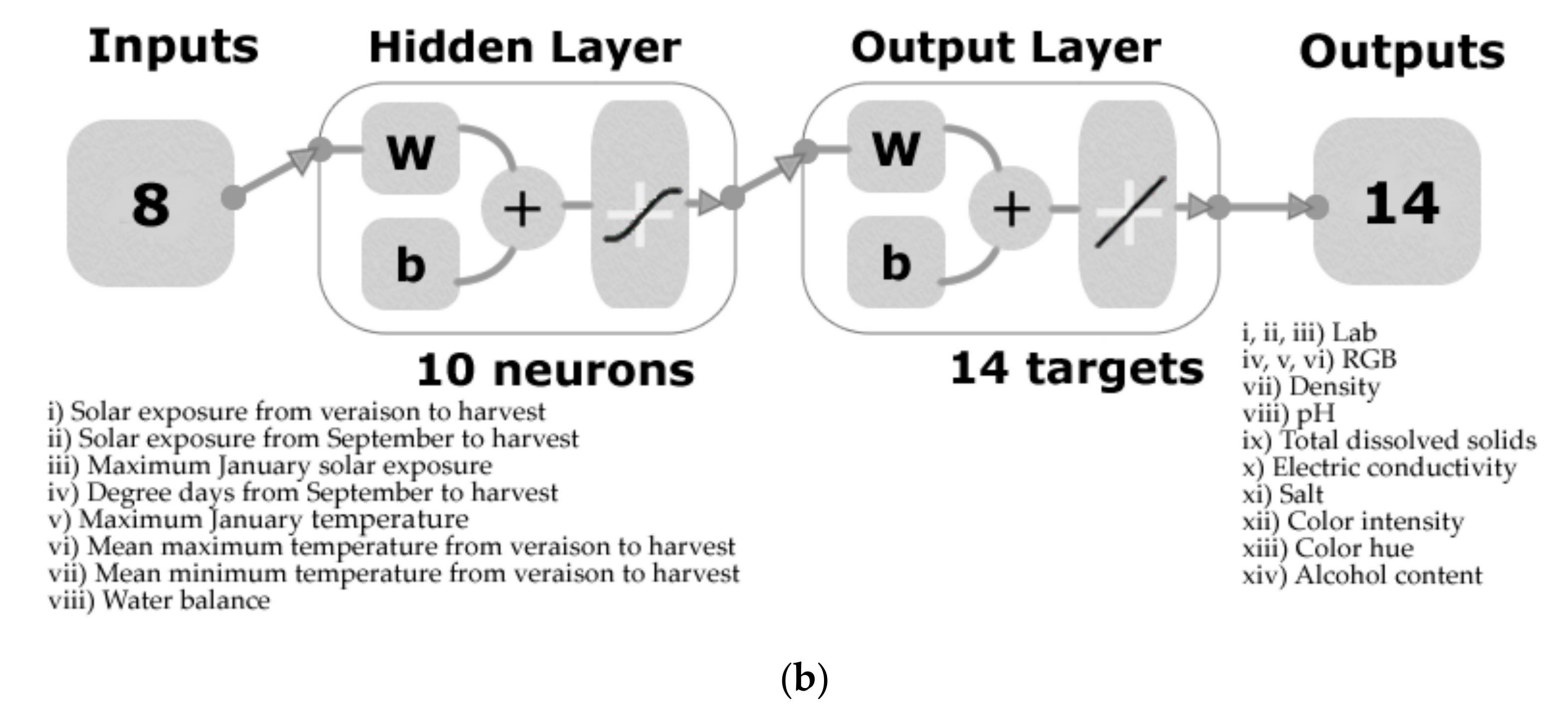
Artificial neural network model diagrams showing the inputs and target/outputs of (**a**) Model 1 to predict the aroma profile based on the peak area of volatile aromatic compounds, and (**b**) the physicochemical data of Pinot noir wines.

Data from weather, physicochemical, and aroma profile measurements were analyzed using a customized code written in Matlab^®^ R2019a (Mathworks, Inc. Natick, MA. USA) to assess significant correlations (*p* < 0.05) between parameters were reported in a matrix. These data were also used to develop machine learning models based on artificial neural networks (ANN) using an automated code in Matlab^®^ that tests 17 different training algorithms in a loop. The weather data related to (i) solar exposure V-H, (ii) solar exposure from S-H, (iii) MJSE, (iv) DD-S-H, (v) MJT, (vi) MeanMaxTV-H, (vii) MeanMinTV-H, and (viii) water balance were used as inputs for machine learning purposes. Two models were developed using these inputs to predict (i) the peak area of nine volatile aromatic compounds measured using the GC-MSD (Model 1) and (ii) 14 physicochemical measurements (Model 2). Both models were developed using normalized data (inputs and targets) from −1 to 1, and with a random data division with 60% of the samples used for training with a Levenberg–Marquardt algorithm, 20% for validation with a mean squared error performance algorithm, and 20% for testing with a default derivative function. The number of neurons was defined by performing a trimming exercise with three, five, seven, and 10 neurons, with 10 neurons giving the best models that contribute to the absence of overfitting. The models consisted of a two-layer feedforward network with a tan-sigmoid function in the hidden layer and a linear transfer function in the output layer (Figure 2).

## 3. Results

Table 1 shows the mean values of the weather data for the vintages with contrasting water balance data (2011–2014). It can be observed that 2011 was the wettest season with the lowest solar exposure and mean temperatures (MeanMaxTV-H and MeanMinTV-H), while 2013 was the driest with the highest MJSE and solar exposure. Vintages 2012 and 2014 presented values in the mid-range.

Table 2 shows the nine volatile compounds identified in all the wine samples tested and the aromas associated with them. It can be observed from this table that most of the aromas are related to fruity scents, especially apple, with two specific compounds (phenylethyl alcohol and ethyl laurate) with floral and one (ethyl palmitate) with milky or creamy notes.

**Figure 3:**
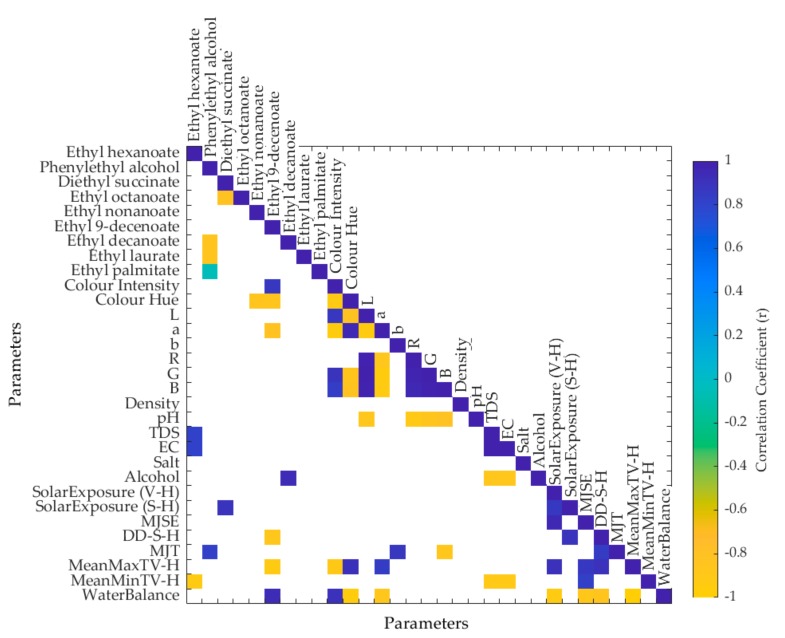
Matrix showing only the significant correlations (*p* < 0.05) between the weather and physicochemical data and volatile aromatic compounds of Pinot noir wines of vintages from 2008 to 2016. Abbreviations: TDS = total dissolved solids, EC = electric conductivity, V-H = veraison to harvest, S-H = September to harvest, MJSE = maximum January solar exposure, DD = degree days, MJT = maximum January temperature, MaxTV-H = maximum temperature veraison to harvest, MinTV-H minimum temperature veraison to harvest.

Figure 3 shows the significant (*p* < 0.05) correlations between the weather information, the aromas, and physicochemical data. It can be observed that the solar exposure from September to harvest was positively correlated with diethyl succinate (*r* = 0.90), while the degree days from September to harvest was negatively correlated with ethyl-9-decenoate (*r* = 0.88). The MJT had a positive correlation with phenylethyl alcohol (*r* = 0.82) and “b” (*r* = 0.88), and a negative correlation with “B”. The MeanMaxTV-H was negatively correlated with ethyl-9-decenoate (*r* = −0.93) and color intensity (*r* = −0.90), as well as positively correlated with color hue (*r* = 0.92) and “a” (*r* = 0.84). On the other hand, the MeanMinTV-H had a negative correlation with ethyl hexanoate (*r* = −0.93), TDS (*r* = −0.90), and EC (*r* = −0.90). Water balance was positively correlated with ethyl-9-decenoate (*r* = 0.93) and color intensity (*r* = 0.90), and negatively correlated with color hue (*r* = −0.95) and “a” (*r* = −0.86). Mean values of the aromatic volatile compounds and physicochemical data are shown as supplementary material in Table S1.

In Table 3, the statistical results from the ANN models are shown. Model 1 had an overall high correlation coefficient (*r* = 0.99) with similar results for all stages (training, validation, and testing; *r* > 0.97) to predict the peak area of nine volatile aromatic compounds (Table 2). From the performance, it can be observed that both validation and testing mean square error (MSE) values were the same (MSE = 0.03), and the training had a lower result (MSE = 0.003), which contributes to the absence of overfitting of the model. Furthermore, the slope (b) for all stages and the overall model was close to the unity (*b* = 0.97). On the other hand, Model 2 had an overall correlation *r* = 0.94 to predict 14 physicochemical parameters (Figure 2b). The slopes from the models of the three stages were high enough (*b* > 0.83) with an overall model *b* = 0.90. Similar to Model 1, the performance of the training stage from Model 2 was lower (MSE = 0.02) than the validation and testing stages, with the last two presenting similar results (MSE = 0.05 and MSE = 0.06; respectively).

**Figure 4:**
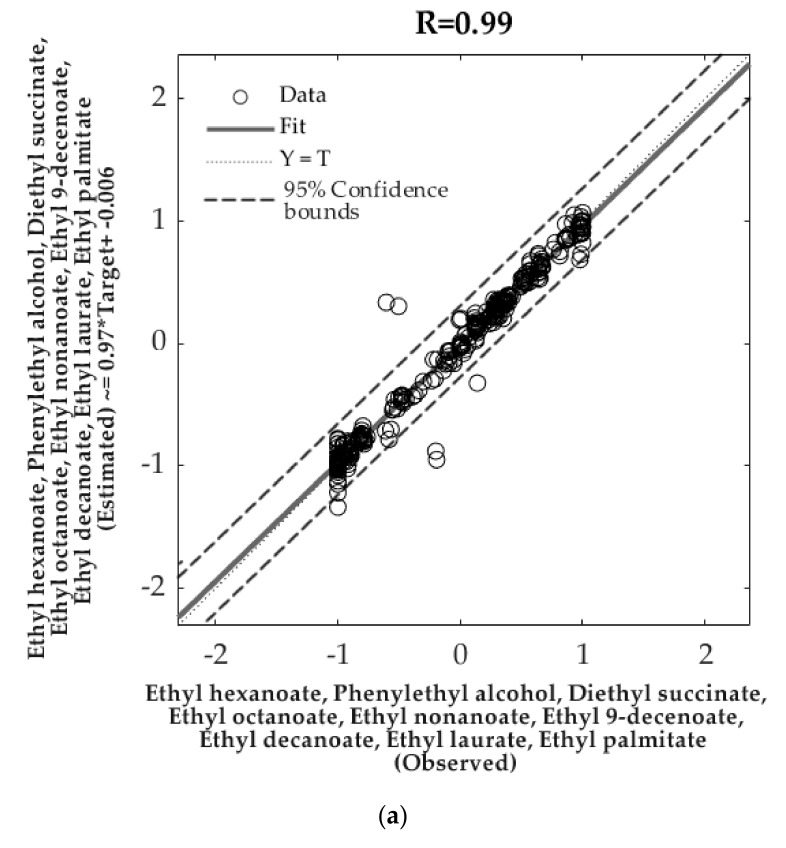

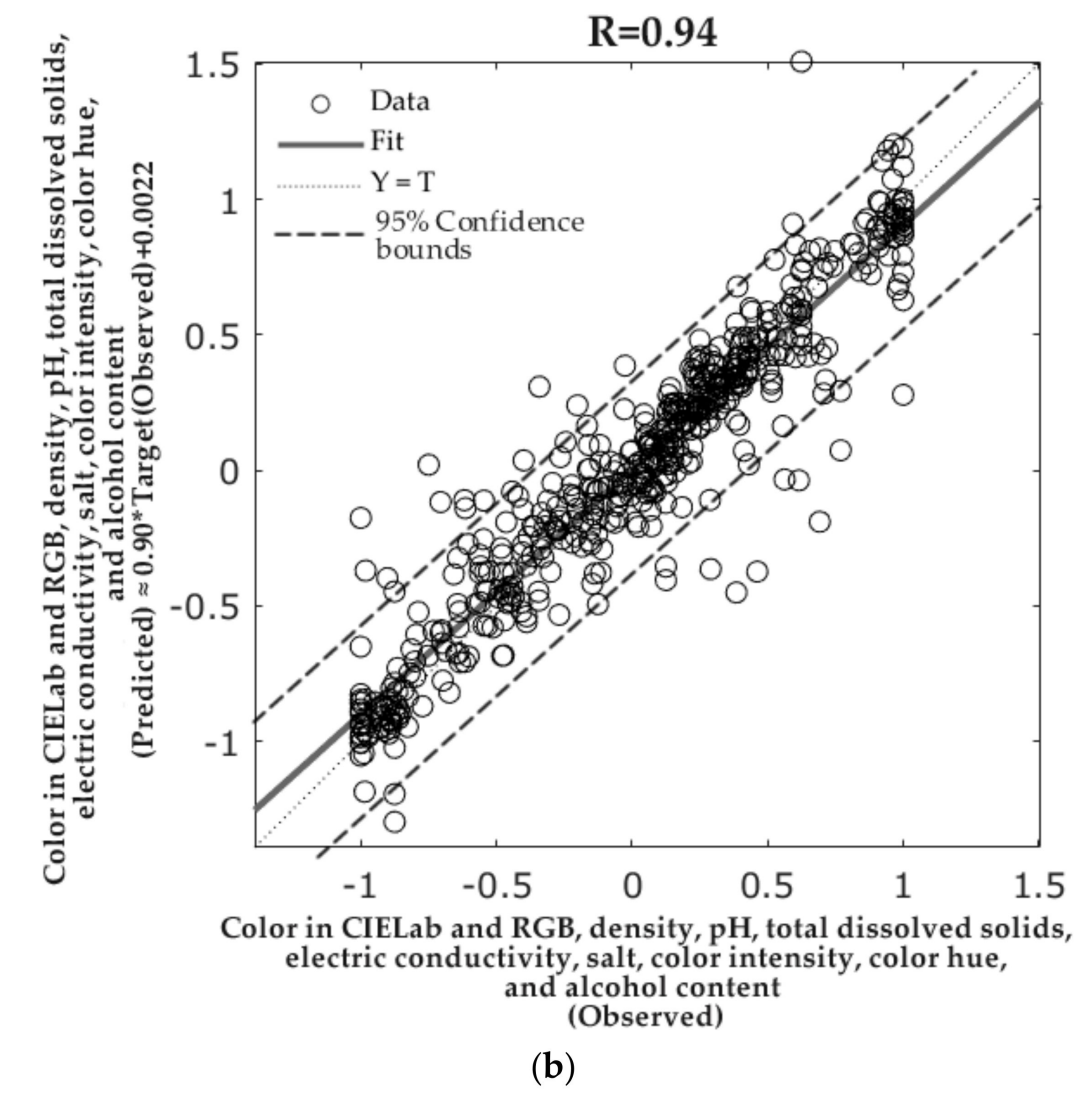
Overall artificial neural network models to predict (**a**) the aroma profile (Model 1) and (**b**) the physicochemical parameters of Pinot noir wines (Model 2), both using the weather data as inputs (Figure 2). The models show the observed (x-axis) and predicted (y-axis) data as well as the 95% confidence bounds.

Figure 4a shows the overall Model 1 to predict the aroma profile based on the peak area of volatile aromatic compounds of Pinot noir wines. From the 95% confidence bounds, only 1.01% of outliers (six out of 594) were found. On the other hand, Figure 4b depicts the overall Model 2 to predict the physicochemical data of the wines. Regarding the 95% prediction bounds, the model presented 3.25% (30 out of 924) of outliers. For both models, several retraining attempts were performed, obtaining similar results to those presented in Table 3 and Figure 4. When feeding these models with new data, the outputs values are given normalized from −1 to 1; however, the reverse function for normalization in Matlab^®^ R2019a (Mathworks Inc., Natick, MA, USA) provides the actual values in the corresponding units.

## 4. Discussion

The physicochemical parameters assessed in this study have been associated with wine quality by other authors. Aromas and color-related parameters are some of the factors that have been the most associated with wine quality [42,43]. Sáenz-Navajas et al. [44] found that there is a relationship between red wine color and the quality perception from consumers and concluded that darker wines with higher red and lower yellow values were rated as higher quality. Jackson et al. [42] reported a significant and positive correlation between both pH and color and overall wine quality. The importance of TDS, EC, and salt measurements rely on the fact that these are an approach to minerals content [45], which are important in wine quality, as the minerals present in wine have been related to those present in the soil, and these have been associated with the wine’s nutritional composition and safety [46].

There was a significant variability within the vintages and the particular region in Victoria analyzed in this study. The extremes can be considered for low-quality wines produced in the 2010–2011 vintage due to heavy rains before harvest, which negatively affects the quality traits in berries and wine [47,48]; this low-quality assessment was obtained from anecdotal information from points received in those particular years and the sensory analysis conducted by the vineyard studied. On the contrary, dry seasons were found for example in 2013–2014 and 2014–2015, with increased berry quality traits that were passed to the respective wines. The latter were mainly due to some control of the water received by plants from irrigation and water deficits. These differences contribute to the robustness of the machine learning models found, which presented no indication of overfitting with high precision in the prediction of the peak area of volatile aromatic compounds (Model 1) and physicochemical wine characteristics (Model 2).

The effects of solar exposure and canopy architecture (which is dependent on water balance) on the aroma profiles of wines have been previously reported, and they are consistent with the data presented in Figure 3. Specifically, these effects manifest through the influence of the microclimate within bunches [49], phenolic compounds [50,51], and the flavonol profile [52]. Due to the direct effect of bunch exposure to radiation in the aroma profiles obtained in wines, researchers have investigated the effect of defoliation as a management strategy to increase berry quality and aroma traits, which depends on the cultivar, timing of defoliation, and climatic region [53,54,55,56,57,58,59,60]. These researches demonstrate the importance of fruit exposure to solar radiation and microclimate conditions that are favorable to the development of berry quality traits.

As previously mentioned, seasonal temperatures not only influence the occurrence and length of different phenological stages in grapevines, such as budbreak, flowering, berry set, pea size, veraison, and harvest, but also the chemical and aroma composition of berries. Of critical importance is the influence of weather parameters, such as temperature [61,62,63,64], and water availability from veraison onwards in red cultivars, which is determinant to the final wine quality and aroma profiles. Several studies have focused on the pre and post veraison phenological stages for irrigation treatments to increase berry and wine quality traits, especially in red cultivars [65,66,67,68,69].

For machine learning modeling, it has been demonstrated that the implementation of important parameters as inputs that directly influence the targets proposed render more robust models in contrast to the usage of raw data. Based on calculated parameters rather than raw data inputs, there are recent studies implementing machine learning to assess beer quality [70,71,72], interpret remote sensing data for plant water status assessment in vineyards [73], chocolate quality assessment by consumers using NIR [74], and aroma profiles in cocoa trees based on canopy architecture parameters [75]. In this study, relevant parameters from weather conditions, management strategies, and physicochemical parameters of wines were obtained and considered as inputs in the machine learning modeling, which can explain the high accuracy obtained for the predictions of Models 1 and 2 without signs of overfitting.

The use of ANN for modeling has the advantage of being able to use multiple targets, which makes the models more efficient. This is due to the easiness of feeding only one model to obtain all the output data instead of having to add the new inputs to several single-target models. Several studies related to food and agriculture have used this type of machine learning algorithms with high performance and accuracy [38,71,72,75,76,77].

The technique proposed considers the readily available weather information from vintages close to the vineyards and a vertical vintage library, which most wineries can obtain easily. The models developed assume that the vineyard management is consistent throughout the seasons, including the winemaking techniques and yeast used. The implementation of these models to other cultivars, environments, and regions will need the incorporation of further site-specific data as inputs and wine chemical and aroma profile analysis from available and contrasting vintages. The latter benefit from the learning aspect of the models proposed, which does not require a full development of new analyses for different regions.

## 5. Conclusions

Artificial intelligence techniques can be implemented in the wine industry from readily available weather and management practices data to assess quality traits in final wines. Modeling strategies using artificial neural networks developed for particular regions can be implemented for other cultivars, environments, and regions by including extreme values from their respective vintages. High accuracy models to determine the aroma profile of wines before the winemaking process can offer a powerful tool to growers and winemakers for the decision making in the vinification process to maintain or increase wine quality and styles. Further research is required to adapt these techniques to canopy management strategies and within-season modeling that can be implemented in real-time within the season to manipulate the final wine and aroma profiles to specific targets using management strategies, such as canopy, fertilization, and irrigation management.

## Figures and Tables

**Table 1:** Mean values of weather data only for the contrasting vintages based on water balance.

Year	Solar Exposure (V-H; MJ m^2 −1^)	Solar Exposure (S-H; MJ m^2 −1^)	MJSE (MJ m^2 −1^)	DD-S-H (days)	MJT (°C)	MeanMaxT V-H (°C)	Mean MinTV-H (°C)	Water Balance (mm)
2011	15.6	19.1	24.6	1066.8	18.6	19.7	9.44	673.7
2012	17.9	20.2	26.3	1147.3	19.4	22.6	10.75	255.9
2013	21.8	21.8	28.9	1234.2	19.8	26.1	12.05	−117.5
2014	19.0	20.0	27.6	1223.7	20.3	25.8	11.31	−61.9

Abbreviations: V-H = veraison to harvest, S-H = September to harvest, MJSE = maximum January solar exposure, DD = degree days, MJT = maximum January temperature, MaxTV-H = maximum temperature veraison to harvest, MinTV-H minimum temperature veraison to harvest.

**Table 2:** Volatile compounds identified using gas chromatography–mass spectroscopy and their associated aromas.

Volatile Compound	Aroma *
Ethyl hexanoate	Apple/Green banana/Pineapple
Phenylethyl alcohol	Rose/Bread/Honey
Diethyl succinate	Cooked apple
Ethyl octanoate	Apple/Banana/Pineapple
Ethyl nonanoate	Cognac/Apple/Winey/Nutty
Ethyl-9-decenoate	Fruity/Fatty/Roses
Ethyl decanoate	Waxy/Apple/Grape
Ethyl laurate	Floral/Soapy/Sweet
Ethyl palmitate	Waxy/Fruity/Creamy/Milky

* The association between the volatile compounds and aromas were obtained from The Good Scents Company [39], Genovese et al. [40], Arcari et al. [41], and Gonzalez Viejo et al. [38].

**Table 3:** Statistics from the artificial neural network models to predict the aroma profile based on the peak area of volatile aromatic compounds (Model 1) and the physicochemical data (Model 2) from Pinot noir wines.

Stage	Samples	Observations	*R*	Slope (b)	Performance (MSE)
**Model 1**					
Training	40	360	0.99	0.98	0.003
Validation	13	117	0.97	0.98	0.03
Testing	13	117	0.97	0.92	0.03
**Overall**	**66**	**594**	**0.99**	**0.97**	/
**Model 2**					
Training	40	560	0.96	0.91	0.02
Validation	13	182	0.93	0.83	0.05
Testing	13	182	0.90	0.94	0.06
**Overall**	**66**	**924**	**0.94**	**0.90**	/

Abbreviations: *R* = correlation coefficient and MSE = mean square error.

## Supplementary Materials

The following are available online at https://www.mdpi.com/2304-8158/9/1/33/s1, Table S1: Means and standard error (SE) of the volatile aromatic compounds and physicochemical parameters of the wined from each vintage.
