# Supplementary material for: Modeling Pinot Noir Aroma Profiles Based on Weather and Water Management Information Using Machine Learning Algorithms: A Vertical Vintage Analysis Using Artificial Intelligence
Source: Foods. 2019 Dec 30;9(1):33. doi: 10.3390/foods9010033 (PMC7023421; doi:10.3390/foods9010033)
Supplement: Supplementary file 1 [file foods-09-00033-s001.pdf]

Table 1. Means and standard error (SE) of the volatile aromatic compounds and physicochemical parameters of the wined from each vintage.

Parameter	2008	2009	2010	2011	2012	2013	2014	2015	2016
**Ethyl hexanoate**	6029895 ± 61086	5203253 ± 100230	4376611 ± 139374	7038112 ± 74282	5856425 ± 990	5512491 ± 48269	6078913 ± 26556	5716304 ± 108335	4221613 ± 24387
**Phenylethyl alcohol**	6826655 ± 159224	5822943 ± 229624	4819232 ± 300023	4910750 ± 83963	3763249 ± 1881625	5176013 ± 2588007	5888210 ± 193981	5859749 ± 81216	6781535 ± 147651
**Diethyl succinate**	15553884 ± 492846	14820676 ± 402260	14087469 ± 311673	9367011 ± 35170	12883311 ± 46636	13447329 ± 166504	9114627 ± 32843	8756200 ± 73448	11012970 ± 204965
**Ethyl octanoate**	29957347 ± 4955524	30070217 ± 2970063	30183086 ± 984602	36843905 ± 401878	35136554 ± 184115	36318374 ± 246762	37710437 ± 315668	37567039 ± 589725	31165578 ± 455310
**Ethyl nonanoate**	0 ± 0	163194 ± 81597	326388 ± 163194	382230 ± 9581	0 ± 0	0 ± 0	0 ± 0	0 ± 0	0 ± 0
**Ethyl 9-decenoate**	0 ± 0	0 ± 0	0 ± 0	1811059 ± 87102	713721 ± 63443	393084 ± 196542	0 ± 0	453005 ± 25338	0 ± 0
**Ethyl decanoate**	12547607 ± 4695216	16796141 ± 2573688	21044676 ± 452159	15944349 ± 622570	21324594 ± 150624	18418270 ± 214950	17716449 ± 167106	18303860 ± 470755	14175413 ± 706036
**Ethyl laurate**	743735 ± 290195	2007095 ± 379109	3270455 ± 468023	1680900 ± 281809	3983444 ± 339138	1614782 ± 323200	979486 ± 33954	935439 ± 31016	1384188 ± 269729
**Ethyl palmitate**	0 ± 0	0 ± 0	0 ± 0	0 ± 0	503503 ± 251752	0 ± 0	0 ± 0	0 ± 0	349778 ± 27943
**Colour Intensity (Au)**	1.26 ± 0.01	1.28 ± 0.02	1.31 ± 0.03	1.57 ± 0.01	1.42 ± 0.02	1.24 ± 0.03	1.10 ± 0.05	1.06 ± 0.03	0.89 ± 0.01
**Colour Hue (unitless)**	0.18 ± 0.001	0.18 ± 0.001	0.18 ± 0.001	0.17 ± 0.002	0.18 ± 0.0.003	0.18 ± 0.001	0.19 ± 0.002	0.18 ± 0.005	0.18 ± 0.004
**L (unitless)**	38.35 ± 0.60	44.38 ± 0.70	50.41 ± 0.79	59.23 ± 0.62	51.98 ± 0.38	50.40 ± 0.73	32.05 ± 0.69	54.76 ± 0.74	47.97 ± 0.68
**a (unitless)**	31.98 ± 0.45	28.71 ± 0.31	25.45 ± 0.16	18.11 ± 0.09	26.44 ± 0.46	29.65 ± 0.74	37.13 ± 1.26	26.29 ± 0.45	35.11 ± 0.67
**b (unitless)**	20.57 ± 0.20	17.29 ± 0.18	14.01 ± 0.17	12.17 ± 0.29	13.31 ± 0.47	14.43 ± 0.88	12.46 ± 0.47	8.20 ± 0.04	5.63 ± 0.38
**R (unitless)**	144.33 ± 2.03	155.83 ± 2.18	167.33 ± 2.33	180.33 ± 1.45	173.00 ± 0.58	173.33 ± 2.91	131.00 ± 3.21	178.33 ± 2.60	170.33 ± 2.67
**G (unitless)**	67.33 ± 1.20	85.00 ± 1.43	102.67 ± 1.67	130.67 ± 1.76	106.00 ± 1.15	99.00 ± 1.53	46.67 ± 1.45	113.33 ± 1.45	89.00 ± 1.53
**B (unitless)**	59.00 ± 1.53	78.17 ± 1.93	97.33 ± 2.33	122.33 ± 1.86	102.33 ± 1.45	97.00 ± 2.08	58.00 ± 1.53	118 ± 2.08	105.67 ± 1.76
**Density ($g \cdot mL^{-1}$)**	0.99 ± 0.01	0.98 ± 0.01	0.98 ± 0.01	0.99 ± 0.002	0.99 ± 0.01	0.98 ± 0.01	0.99 ± 0.01	0.99 ± 0.01	1.00 ± 0.01
**pH (unitless)**	3.70 ± 0.05	3.57 ± 0.02	3.70 ± 0.05	3.60 ± 0.04	3.60 ± 0.03	3.60 ± 0.04	3.80 ± 0.04	3.70 ± 0.04	3.50 ± 0.03

**Total dissolved solids (ppm)**	909<br>± 6.11	865<br>± 7.37	820<br>± 8.63	932<br>± 5.80	842<br>± 7.12	820<br>± 6.98	857<br>± 5.29	842<br>± 8.73	849<br>± 10.61
**Electric conductivity (µs·cm^{-1})**	1934<br>± 13.81	1839<br>± 10.80	1744<br>± 7.79	1982<br>± 32.50	1791<br>± 23.58	1744<br>± 13.64	1823<br>± 23.82	1791<br>± 17.38	1806<br>± 6.43
**Salt (%)**	0.08<br>± 0.001	0.07<br>± 0.001	0.06<br>± 0.001	0.07<br>± 0.000	0.07<br>± 0.000	0.06<br>± 0.000	0.07<br>± 0.001	0.07<br>± 0.001	0.07<br>± 0.001
**Alcohol (%)**	12.60<br>± 0.00	13.90<br>± 0.00	13.90<br>± 0.03	12.70<br>± 0.00	14.20<br>± 0.00	13.60<br>± 0.03	13.60<br>± 0.00	14.20<br>± 0.00	13.00<br>± 0.00

*All volatile aromatic compounds are reported as peak areas.
